# 3-Arylcoumarin inhibits vascular calcification by inhibiting the generation of AGEs and anti-oxidative stress

**DOI:** 10.1080/14756366.2022.2109024

**Published:** 2022-08-11

**Authors:** YuFei Li, Yinbo Pan, Liying Wang, Xiaojing Wang, Haiping Chu, Yan Li, Yanling Mu, Jie Sun

**Affiliations:** a Department of Anesthesiology and Perioperative Medicine, The First Affiliated Hospital of Shandong First Medical University & Shandong Provincial Qianfoshan Hospital, Jinan, China; b Institute of Materia Medica, Shandong First Medical University & Shandong Academy of Medical Sciences, Jinan, China; c Shandong Electric Power Central Hospital, Jinan, China

**Keywords:** 3-arylcoumarin derivatives, vascular calcification, AGEs, oxidative stress

## Abstract

**Objective:**

This work aims to screen drugs for preventing and treating vascular calcification. **Method:** We screened a series of 3-arylcoumarins for the detection of vascular calcification-associated factors using human aortic vascular smooth muscle cells.

**Results:**

We found that compounds **14** and **32** significantly inhibited alkaline phosphatase (ALP) activity similar to aminoguanidine hydrochloride (AGH) in a cellular model of AGEs-induced calcification. We also found that compounds **14** and **32** could significantly decrease the levels of factors such as AGEs, intracellular calcium ions, and total ROS in the calcified cell model. Further study indicates that compound **14** could significantly inhibit the expression of P-ERK1/2, PKC, NF-κB, RAGE and OPN proteins and increased the expression of SM22-α and PPAR-γ proteins in the calcified cells.

**Conclusion:**

We speculate that compound **14** inhibits vascular calcification by inhibiting oxidative stress and inhibiting AGEs production, suggesting that 3-arylcoumarin derivatives are potential candidates for the treatment of vascular calcification.

## Novelty Statement

Vascular calcification is a process similar to bone formation, which is highly adjustable and active. Currently, there are no specific drugs to delay or reverse vascular calcification. Through the screening of 44 coumarin compounds synthesised by our group, compound **14** was obtained to dose-dependently inhibit the calcification of vascular smooth muscle cells without affecting the normal proliferation of cells, decreasing the intracellular calcium concentration, inhibiting the activity of ALP enzyme. In conclusion, the calcium lowering effect of compound **14** is a potential candidate for drugs for the treatment of vascular calcification.

## 1. Introduction

Vascular calcification is a process similar to bone formation, which is highly adjustable and active^1^^,^^2^. Its occurrence is related to the phenotypic transformation from human aortic smooth muscle cells (HASMCs) to osteoblasts caused by various stimulating factors such as hyperglycemia^3^^,^^4^, oxidative stress^5^ and inflammatory response^6^.

Vascular calcification (VC), a characteristic of advanced atherosclerosis, is an ectopic accumulation of calcium phosphate in vascular tissue. VC increases dramatically in patients with diabetes and chronic kidney disease. Severe atherosclerosis with massive vascular calcification is a key determinant of increased cardiovascular mortality, and it is thought that an active process is controlled by highly regulated cellular and molecular pathways, similar to those involved in bone formation. ALP is one of the first functional genes in the calcification mechanism^7^. ALP is highly expressed in the cells of calcified tissue and plays a critical function in the formation of hard tissue. ALP increases inorganic phosphate local rates and facilitates calcification as well as reduces the extracellular pyrophosphate concentration, an inhibitor of mineral formation. The mechanisms involved in vascular calcification are complex and relatively poorly understood. At the same time, because arterial calcification has no effective measures in the current clinical treatment, it is difficult to reverse it whether it is conventional drugs as well as vascular interventional means or surgery. At present, vascular calcification has become a growing cardiovascular problem.

Some available drugs can only reduce the body's blood lipids and blood glucose levels by reducing the oxidative stress response associated with calcification and blocking the signalling molecules and pathways of the inflammatory response, for example, statins delay the progression of vascular calcification by lowering blood lipid levels and metformin by lowering blood glucose^8–12^. Taking advantage of existing drug targets to improve the design and development of vascular calcification drugs appears to be important.

More and more studies have shown that, compared with single-target drugs, multi-target natural medicines have irreplaceable advantages in cardiovascular protection ^13–15^. For example, coumarin compounds have efficient AGEs inhibitory activity^12^, and isoflavone compounds are effective antioxidants^16^. It is believed that if a class of drugs can be designed to target both AGEs and oxidative stress it will have advantages in the field of vascular protection to achieve a better effect of treatment.

Previously, our research group designed and synthesis more than 500 arylcoumarins, and it was found that 44 compounds showed appreciable activities through screening experiments *in vitro*^17^. In this paper, we will further investigate to find out the compounds that have the activity of inhibiting vascular calcification, and study the related mechanism.

## 2 Materials and methods

### 2.1. Materials

#### 2.1.1. The following cells were used for culture

HASMCs were provided by the Institute of Materia Medica, School of Pharmacy and Pharmaceutical Sciences, Shandong First Medical University. Using 10% FBS-containing DMEM culture medium for culture (Table 1).

#### 2.1.2. The following reagent were used

0.2% Alizarin Red S stain solution (Solarbio, China), Foetal Bovine Serum (Gbico, USA), Alkaline Phosphatase Activity Test Kit (Beyotime, China), DMEM Medium (Gbico, USA), WBKLS0100 (Millipore, USA), Calcium ion detection kit (LEAGENE, China), ROS detection kit (Xi'an Baiaolaibo Biotechnology Co., Ltd.), MTT (Solarbio, China), ERK1/2 antibody (Cell Signalling Technology, China), PKC antibody (Cell Signalling Technology, China), RAGE antibody (Immunoway, USA), NF-κB antibody (Cell Signalling Technology, China), β-actin antibody (proteintech, USA), horseradish labelled goat anti-mouse gG (H + L) (proteintech, USA), l horseradish labelled goat anti-rabbit lgG (H + L) (zsbio), ELISA kit (IL-β, IL-6, TNF-α, AGEs Bpro, China), superoxide dismutase (SOD) detection kit (Solarbio, China), total RNA extraction kit (Solarbio, China), total protein extraction kit (Bestbio, China)), Real Time q-PCR kit (Ta Ka Ra) Primers were commissioned by biosune biotechnology (China) to design synthetic primer sequences (Table 2).

**Table 2.** Primer sequence.

Gene	The sequence (5'→3')	Primer length/bp
RUNX2	F： TCTCCAACCCACGAATGCAC	76
	R： ATACCGAGGGACATGCCTGA	

#### 2.1.3. The following instruments were used

Model 3111 CO_2_ incubator (Thermo, USA), Eclipse TE 2000-S fluorescence microscope (Nikon, Japan), Infinite M 200 PRO microplate reader (TECAN, France), CFX96 TOUCK fluorescence quantitative PCR instrument (Bio-Rad, USA), Trans-Blot SD semi-dry transfer system (Bio-Rad, USA), Azure cSeries 200 chemiluminescence imager (Azure Biosystems, USA), Evolution 220 UV spectrophotometer (Thermo, USA).

### 2.2. Methods

#### 2.2.1. Synthesis

**Scheme 1.**
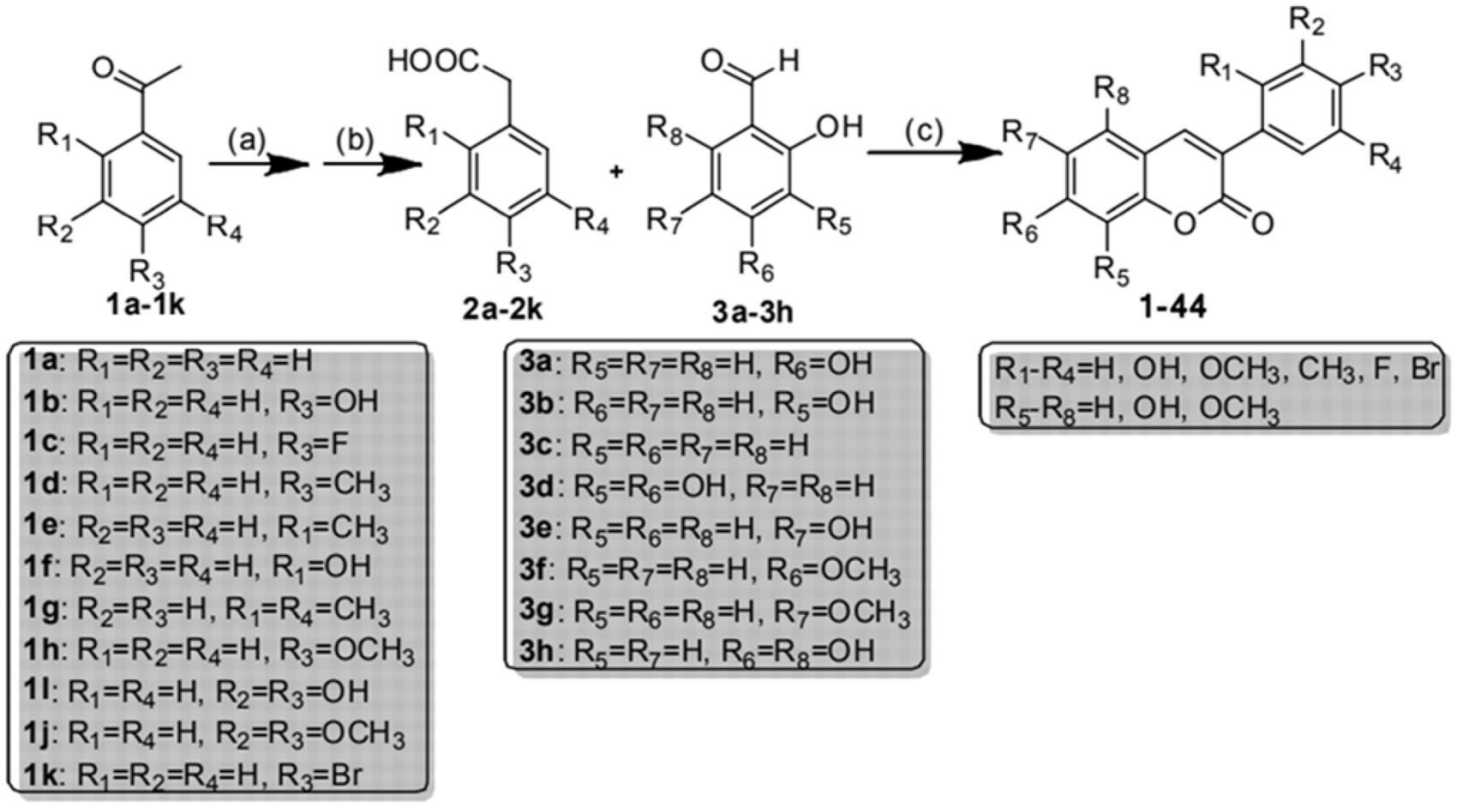
General synthetic route to 3-arylcoumarin derivatives. Reagents and conditions.

The 3-arylcoumarin backbone was synthesised as described in Scheme 1^17^, and its detailed synthesis steps are as follows.

##### 2.2.1.1 Synthesis of compounds 2a–2k

2.40g (20 mmol) of acetophenone, 1.28 g (40 mmol) of sulphur, 6 ml (60 mmol) of morpholine and 0.1 g (0.6 mmol) of p-toluenesulfonic acid are added into a 100 ml three-port reaction flask, heated to 120 °C and refluxed for 8 h. TCL detects the reaction process. The heating is stopped when the raw material point disappears. After the reaction, cool for 10 min, add 50 ml 70% ethanol and 3 ml 50% NaOH solution, heat to 100 °C and reflux for 5 h; after the reaction, pour the reaction solution into 100 ml ice water, stir, and filter to remove insoluble matter. The filtrate was acidified with hydrochloric acid to Ph = 3–4, left overnight, and filtered to obtain a crude white product. The crude product was recrystallized with water to obtain white pure product.

##### 2.2.1.2 Synthesis of compound 3h

Add 6.30 g (50 mmol) of phloroglucinol and 40 ml of ethyl acetate into a 100 ml three-mouth reaction flask, add 10 ml of DMF after it is completely dissolved, continue stirring, add 6 ml (60 mmol) of POCl_3_ dropwise within 10 min under ice bath, stir for 20 min, withdraw the ice bath, stir at room temperature for 1 h, stir for 1 h after off-white precipitate appears, stop the reaction. Add 40 ml water to the reaction solution to dissolve the precipitate, take the organic layer, wash the organic layer with saturated sodium chloride solution 3 times, and dry the anhydrous sodium sulphate overnight. Anhydrous sodium sulphate was removed by suction filtration, and the filtrate was concentrated to obtain 2,4,6-trihydroxybenzaldehyde (3 h), a light red solid. The synthesis of compounds **3a–3g** was performed for 3 h.

##### 2.2.1.3 Synthesis of Compound 1

1.36 g (10 mmol) of phenylacetic acid, 1.38 g (10 mmol) of 2,4 dihydroxybenzaldehyde, 5.56 g (55 mmol) of triethylamine and 6.12 g (60 mmol) of acetic anhydride were added to a 100-ml triple-mouth reaction flask and refluxed for 6 h at 112 °C with stirring (monitored by TLC). After the reaction, pour 50 ml of water while hot, stir, precipitate a large number of solids, allow to stand, filter, wash the filter cake with water until the washing solution is nearly neutral, dry and recrystallize with ethyl acetate/petroleum ether to obtain a yellowish needle-like solid. Add the obtained solid into a 100 ml three-mouth reaction flask, add 10 ml ethanol and heat and stir until the solid is completely dissolved, then add 40 ml 10% hydrochloric acid, stir well, heat to 80 °C and reflux for the reaction for 5 h. After the completion of the TLC monitoring reaction, pour the reaction solution into 50 ml of ice water, stir to precipitate a large amount of solid, withdraw and filter after standing, recrystallize the filter cake with ethanol/water, and dry to the white pure product in a vacuum oven. The synthesis of compounds **2–44** was performed as for 1.

#### 2.2.2. Induction of calcification of human aortic vascular smooth muscle cells

The second to fifth-generation human aortic smooth muscle cells were taken for experiments. The cells were cultured in a DMEM medium containing 10% foetal bovine serum. After cell confluence, the mineralisation induction solution containing 100 mg/L AGEs-BSA was added and cultured for 4 days.

#### 2.2.3. Screen 44 compounds based on the inhibition rate of ALP enzyme activity

Cells were seeded on a 24-well plate at a density of 5.0 × 104 cells/well, and the normal control group (HASMCs normal culture), vehicle control group (HASMCs + 5‰ DMSO), and calcification induction group (HASMCs + 100 mg/L AGEs-BSA), negative control group (HASMCs + 100 mg/L AGEs-BSA +5‰ DMSO), positive control group (HASMCs + 100 mg/L AGEs-BSA +5‰ DMSO + AGH6.25 mg/L), compounds **1–44** In the interference group (HASMCs + 100 mg/L AGEs-BSA + 5‰ DMSO + compound 6.25 mg/L), after the cell culture of each group is completed, it is determined according to the alkaline phosphatase activity determination kit. (Note: Aminoguanidine hydrochloride is an inhibitor of AGEs production. Studies have shown that it can inhibit the glycosylation modification of proteins in the body and has obvious preventive and therapeutic effects on diabetic vascular complications^18^ After the MTT assay, DMSO at this concentration had no significant effect on cell proliferation rate).

#### 2.2.4. Alizarin red staining experiment

After the completion of cell culture in each group, staining was performed according to the instructions of the Alizarin Red Staining Kit, and calcium nodule staining was observed and photographed under a microscope. After staining, perform alizarin red quantification, add 200 μl cetyl pyridinium chloride to each well (preparation method: weigh 0.9 g cetyl pyridinium chloride, dissolve in 9 ml distilled water and mix well), incubate at 37 °C for 1 h, Detect absorbance at 562 nm wavelength, perform three parallel sets of tests, record and analyse data.

#### 2.2.5. Cell survival rate experiment

After the cell culture of each group is completed, the cells are digested with 0.25% trypsin and seeded in a 96-well plate at a density of 5.0 × 10^4^ cells per well. After the culture is attached, the culture medium of each group is replaced with serum-free DMEM. The culture solution is to synchronise the cells, and the grouping and processing are the same as each group with 6 holes, repeated 3 times. The MTT method was used to determine cell proliferation in each group.

#### 2.2.6. Determination of AGEs content

After the cell induction of each group is completed, follow the instructions of the ELISA kit.

#### 2.2.7. Determination of intracellular calcium ion concentration

After the cell culture of each group is completed, take appropriate cells for homogenisation, centrifuge at low speed to take the supernatant, BCA protein concentration determination kit to determine protein concentration, use calcium determination kit (o-cresolphthalein complex copper method) to determine the calcium content of each group of cells.

#### 2.2.8. Determination of total active oxygen content in cells

After the cell culture of each group was completed, the ROS content was detected with a reactive oxygen detection kit, the culture medium was removed, the cells were washed with PBS solution, and the fluorescent dye DCFH-DA was added, incubated for 20 min, then washed with PBS, perform three parallel sets of tests, and photographed under a fluorescence microscope^19^.

#### 2.2.9. Determination of superoxide dismutase content

After the cell culture of each group was completed, the cells were collected into a centrifuge tube, ultrasonically broken and centrifuged, and the SOD content of each group of cells was detected according to the SOD activity determination kit (xanthine oxidase method)^19^.

#### 2.2.10. Determination of inflammatory factor expression

After the cell induction of each group is completed, follow the instructions of the ELISA kit.

#### 2.2.11. Runx2 mRNA expression measurement

After the induction of each group of cells, the total mRNA of each group of cells was extracted, reverse transcribed into complementary deoxyribonucleic acid (cDNA), and real-time fluorescent quantitative PCR was performed using cDNA as a template. Using GAPDH as an internal reference, the fold ratio of Runx2m RNA expression in each group was calculated as the relative expression of Runx2 mRNA in each group.

#### 2.2.12. ERK1/2, PKC, NF-κB, RAGE, SM22-α, OPN, PPAR-γ protein expression

After the cells of each group were induced, they were rinsed twice with pre-cooled PBS. Refer to the operating instructions of the total protein extraction kit to scrape the cells and extract the total protein. After taking the supernatant, the BCA method was used for protein quantification. Take 20 μg protein for SDS-polyacrylamide gel electrophoresis, transfer membrane, block, add rabbit anti-human antibody (T-ERK1/2, P-ERK1/2, PKC, NF-κB, RAGE, SM22-α, OPN, PPAR-γ 1:1000), incubate overnight at 4 °C, add secondary antibody Ig G (1:10000), incubate for 1 h at room temperature, developed using ECL kit, use β-actin as the internal reference for data Standardisation, using the control group as the reference sample to calculate the relative expression level of the target protein in each group.

#### 2.2.13. Molecular docking experiment

The compound was docked using the CDOCKER docking procedure. The BSA crystal structure used in this study is taken from the PDB (Protein Data Bank) structure database (http://www.rcsb.org/pdb), the PDB code (PDB ID) of BSA is 4F5S, and the pre-processed small molecule library the compound molecule of is used as the ligand, and the protein crystal structure of the pre-treated and defined active site is used as the receptor, and the remaining settings are kept as default.

#### 2.2.14. Statistical methods

SPSS 22.0 software was used for data analysis, and the data results were all expressed in terms of mean ± standard deviation (SD). One-way analysis of variance (ANOVA) was used for comparison. *P* < 0.05 indicates a significant difference.

## 3 Results and discussion

### 3.1. Effects of compounds on ALP activity

**Figure 1.**
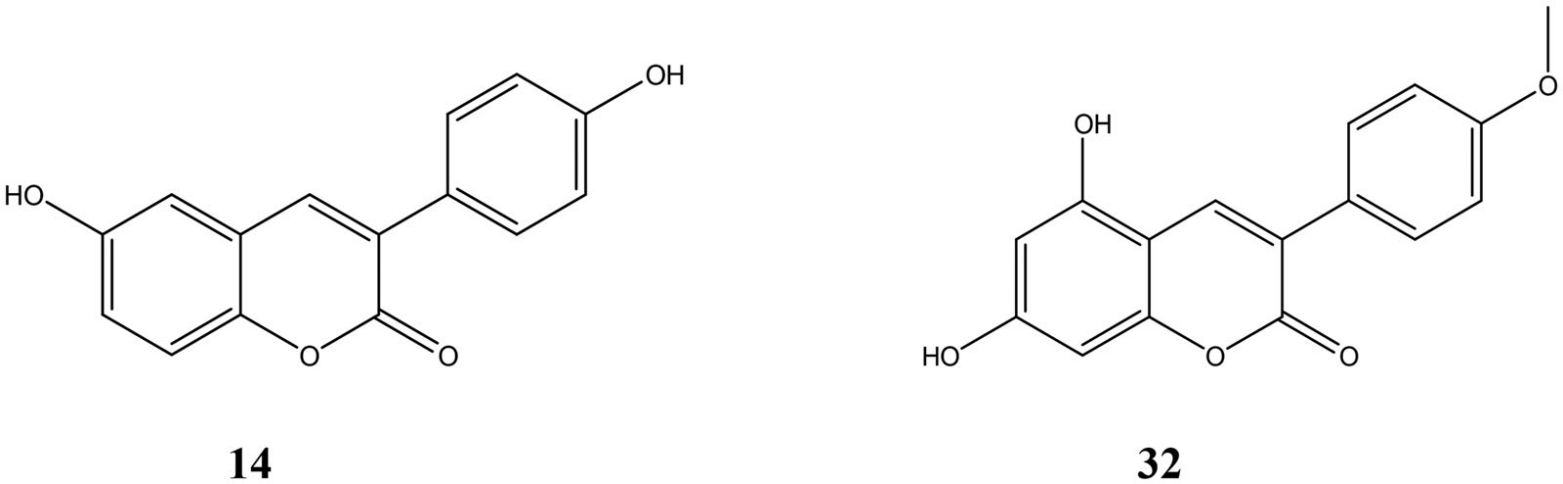
The more active compounds, the structural formulas of **14** and **32**.

Studies have shown that ALP expression is closely related to aortic calcification, and it is a reliable indicator for evaluating vascular calcification and osteoporosis^20^^,^^21^. By measuring the ALP activity in each compound intervention group, we found that most of the compounds can inhibit the increase of ALP induced by AGEs in vascular smooth muscle cells (*P* < 0.05), and compounds **14** (Molecular weight 254.06) and 32 (Molecular weight 284.07) (Figure 1) can significantly inhibit the ALP activity of calcified cells (*P* < 0.01) (Table 3). We speculate that compounds **14** and **32** may have an effective inhibitory effect on calcification. Therefore, we set up corresponding concentration gradients (1.5625 mg/L, 3.125 mg/L, and 6.25 mg/L) for these two groups of compounds, and conducted subsequent *in vitro* activity studies. At these concentrations, the compound was not significantly toxic to the cells.

**Table 3.** Effects of compounds on ALP activity.

Compound	ALP activity (U/mg)
Normal	0.116 ± 0.003
DMSO	0.116 ± 0.003
AGEs	0.240 ± 0.003**
AGEs + DMSO	0.237 ± 0.002**
AGH	0.182 ± 0.003^ΔΔ^
**1**	0.237 ± 0.004
**2**	0.229 ± 0.005^Δ^
**3**	0.228 ± 0.003^ΔΔ^
**4**	0.211 ± 0.005^ΔΔ^
**5**	0.234 ± 0.005
**6**	0.229 ± 0.004^ΔΔ^
**7**	0.214 ± 0.005^ΔΔ^
**8**	0.239 ± 0.003^Δ^
**9**	0.207 ± 0.005^ΔΔ^
**10**	0.207 ± 0.006^ΔΔ^
**11**	0.221 ± 0.002^ΔΔ^
**12**	0.213 ± 0.003^ΔΔ^
**13**	0.204 ± 0.004^ΔΔ^
**14**	0.182 ± 0.003^ΔΔ^
**15**	0.203 ± 0.003^ΔΔ^
**16**	0.229 ± 0.003^ΔΔ^
**17**	0.209 ± 0.003^ΔΔ^
**18**	0.221 ± 0.002^ΔΔ^
**19**	0.220 ± 0.003^ΔΔ^
**20**	0.206 ± 0.003^ΔΔ^
**21**	0.224 ± 0.002^ΔΔ^
**22**	0.230 ± 0.003^ΔΔ^
**23**	0.236 ± 0.003
**24**	0.228 ± 0.003^ΔΔ^
**25**	0.207 ± 0.003^ΔΔ^
**26**	0.234 ± 0.002
**27**	0.237 ± 0.004
**28**	0.206 ± 0.002^ΔΔ^
**29**	0.230 ± 0.006^Δ^
**30**	0.231 ± 0.003^ΔΔ^
**31**	0.214 ± 0.003^ΔΔ^
**32**	0.185 ± 0.002^ΔΔ^
**33**	0.201 ± 0.005^ΔΔ^
**34**	0.207 ± 0.003^ΔΔ^
**35**	0.216 ± 0.016^Δ^
**36**	0.223 ± 0.002^ΔΔ^
**37**	0.205 ± 0.004^ΔΔ^
**38**	0.235 ± 0.002
**39**	0.221 ± 0.003^ΔΔ^
**40**	0.217 ± 0.003^ΔΔ^
**41**	0.221 ± 0.003^ΔΔ^
**42**	0.215 ± 0.004^ΔΔ^
**43**	0.220 ± 0.003^ΔΔ^
**44**	0.205 ± 0.003^ΔΔ^

Effects of compounds on ALP activity. Most of the compounds can inhibit the increase of ALP induced by AGE, in vascular smooth muscle cells, and **14** and **32** have the best inhibitory effect on the ALP activity of calcified cells. ($\bar{x}\pm s,$ *n* = 3, **P < 0.01, compared with normal control group at the same time. ^Δ^*P* < 0.05, ^ΔΔ^*P* < 0.01, compared with AGEs group at the same time).

### 3.2. Effects of compounds 14 and 32 on calcified nodules of smooth muscle cells

**Figure 2.**
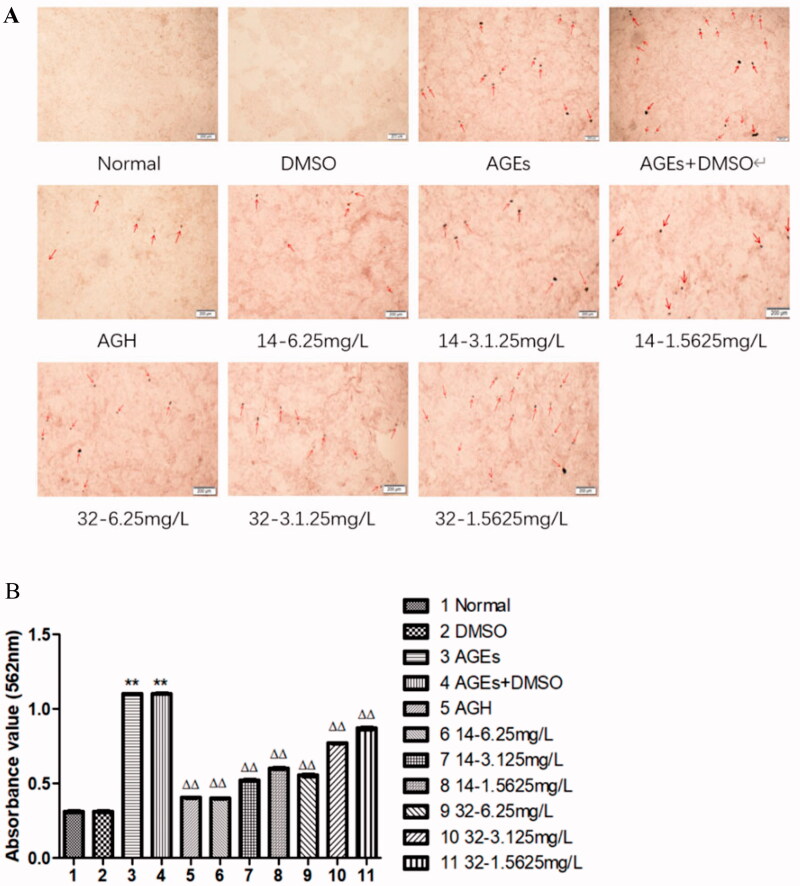
Calcified nodules of smooth muscle cells stained with alizarin red. AGEs can promote the production of calcium nodules in smooth muscle cells, while compounds **14** and **32** can inhibit the production of calcium nodules.

According to the photos taken by the microscope and the quantitative results of Alizarin Red, compared with the control group, the number of intracellular and extracellular calcium nodules in the AGEs group increased significantly (*P* < 0.01). Compared with the AGEs group, treatment with increasing concentrations of compound **14** or **32** significantly reduced the number and area of calcium nodules (*P* < 0.01). In conclusion, compounds **14** and **32** can partially inhibit the calcification in HASMCs induced by AGEs, which is concentration-dependent (Figure 2).

### 3.3. Effects of compound 14 and 32 on cell viability

**Figure 3.**
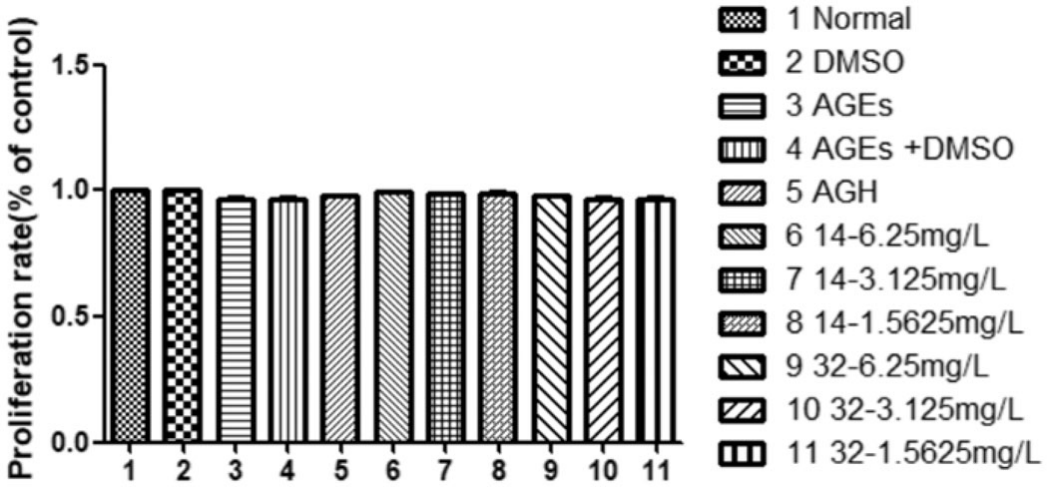
Effects of different concentrations of compounds **14** and **32** on cell viability.

The results of the MTT assay showed that neither AGEs nor compounds **14** or **32** had a significant effect on cell survival *(P > 0.05)* (Figure 3). These results indicate that the compounds are not toxic to cells.

### 3.4. Effects of compound 14 and 32 on AGEs

**Figure 4.**
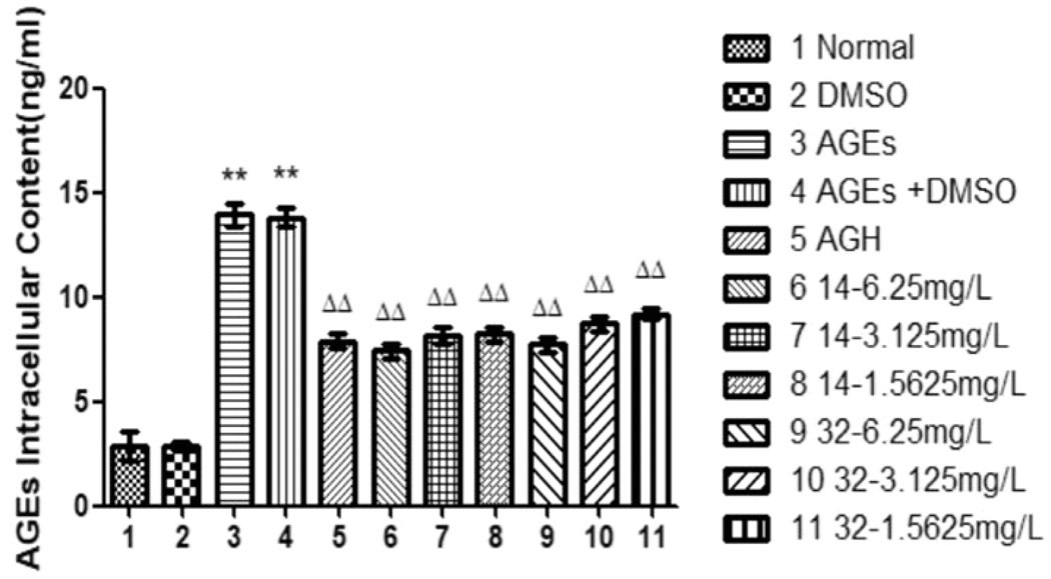
Effects of different concentrations of **14** and **32** on AGEs content. Each compound group can reduce the expression of AGEs.

AGEs can promote calcification of vascular smooth muscle cells^21^^,^^22^. Compared with that in the control group, the intracellular AGEs concentration in the AGEs group was significantly increased (*P* < 0.01) (Figure 4). As the concentration increases, compound **14** or **32** progressively decreases AGEs in the cells (*P* < 0.01). We speculate that compound **14** may promote the cleavage of AGEs, thereby downregulating the expression of RAGE receptors and inhibiting downstream signalling pathways and multiple signal transduction proteins. It ultimately inhibits AGEs-induced differentiation of HASMCs into osteoblasts.

### 3.5. Effects of compound 14 and 32 on intracellular calcium

**Figure 5.**
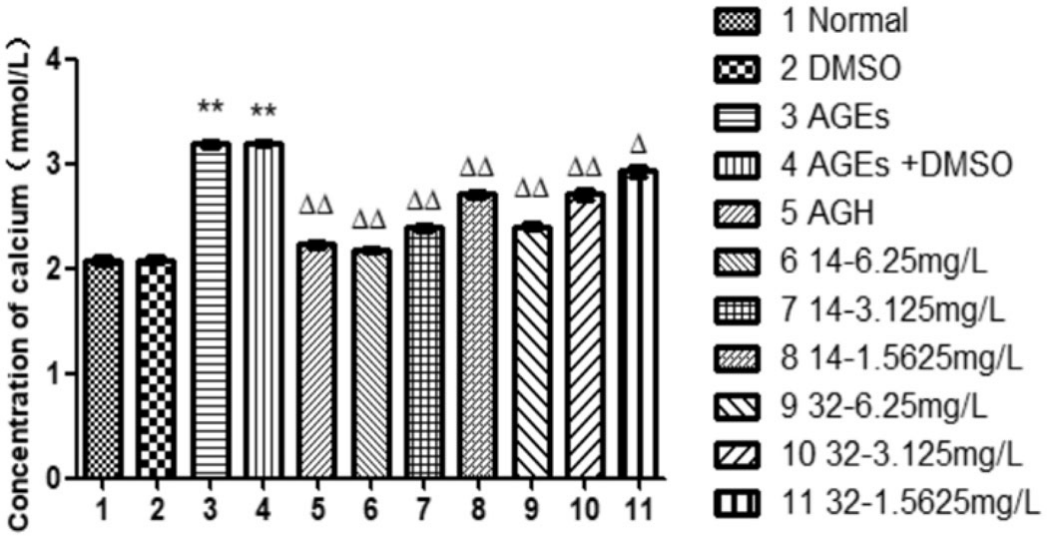
Effects of different concentrations of **14** and **32** on intracellular calcium content.

Compared with that in the control group, the intracellular calcium in the AGEs group was increased significantly (*P* < 0.01), indicating that AGEs can induce calcification of HASMCs. The intracellular calcium in the group of compounds **14** and **32** were progressively reduced at elevated concentrations (*P* < 0.05), suggesting that compounds **14** and **32** can inhibit the calcification of HASMCs induced by AGEs in a concentration-dependent manner (Figure 5). Studies have shown that intracellular calcium and ALP activity are positively correlated with HASMCs calcification, suggesting again that compounds **14** and **32** can inhibit AGEs-induced vascular calcification.

### 3.6. Effects of compound 14 and 32 on ROS

**Figure 6.**
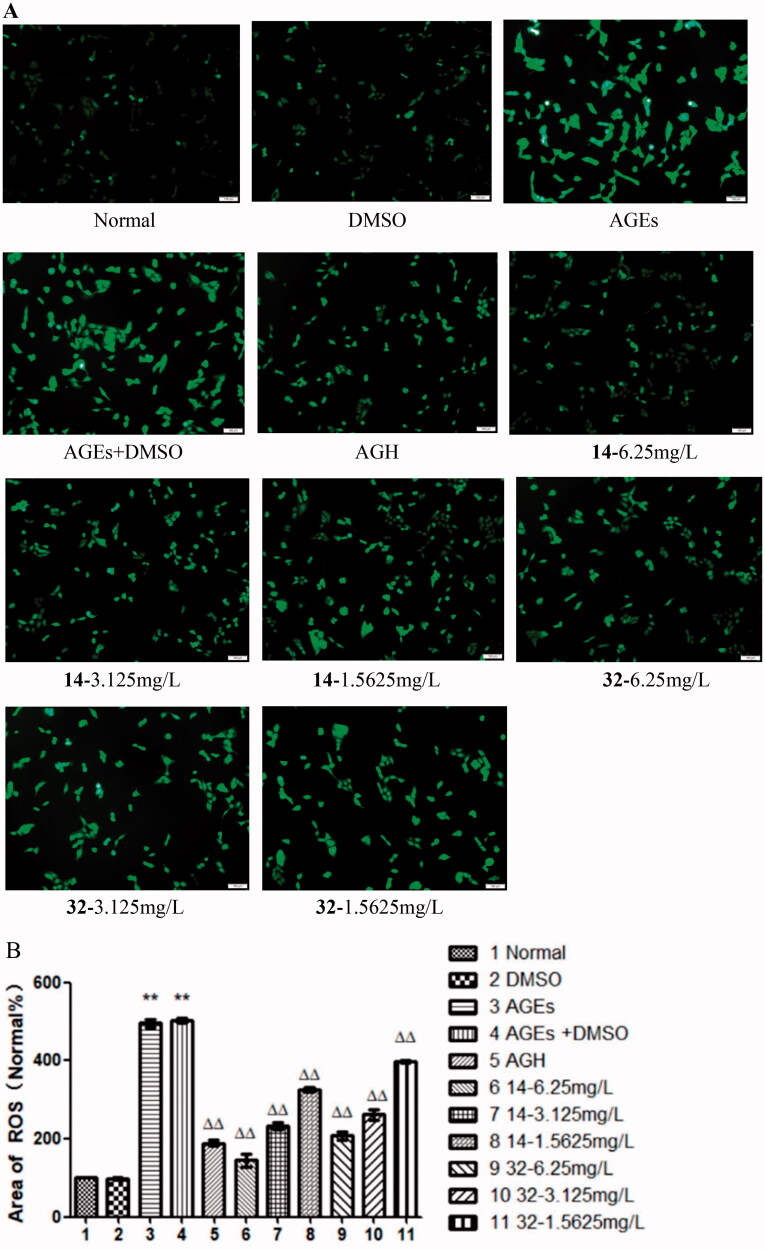
Effects of compounds **14** and **32** on ROS.

Oxidative stress can induce cardiovascular disease^23^. The green fluorescence area for ROS of cells in the AGEs group was significantly increased (*P* < 0.01). In contrast, the green fluorescence area was progressively decreased with elevated concentrations of compounds **14** and **32** (*P* < 0.01). The results indicate that AGEs can induce oxidative stress in HASMCs, while compounds **14** and **32** inhibit this effect and protect HASMCs (Figure 6).

### 3.7. Effects of compound 14 and 32 on SOD expression

**Figure 7.**
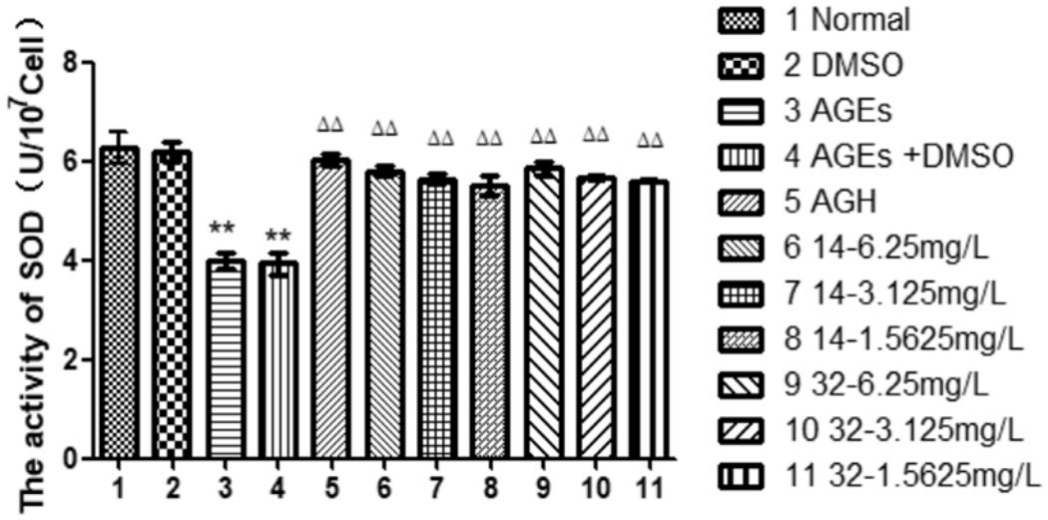
The effect of different concentrations of **14** and **32** on SOD expression.

The activity of the antioxidant index SOD in the AGEs group was significantly reduced (*P* < 0.01). While, the SOD activity in the cells was progressively increased by elevated concentrations of compounds **14** or **32** (*P* < 0.01), suggesting that compounds **14** and **32** can enhance the antioxidant capacity of the cells in a concentration-dependent manner (Figure 7).

### 3.8. The effects of compound 14 and 32 on the expression of inflammatory cytokine TNF-α, 1 L-6, 1 L-β

**Figure 8.**
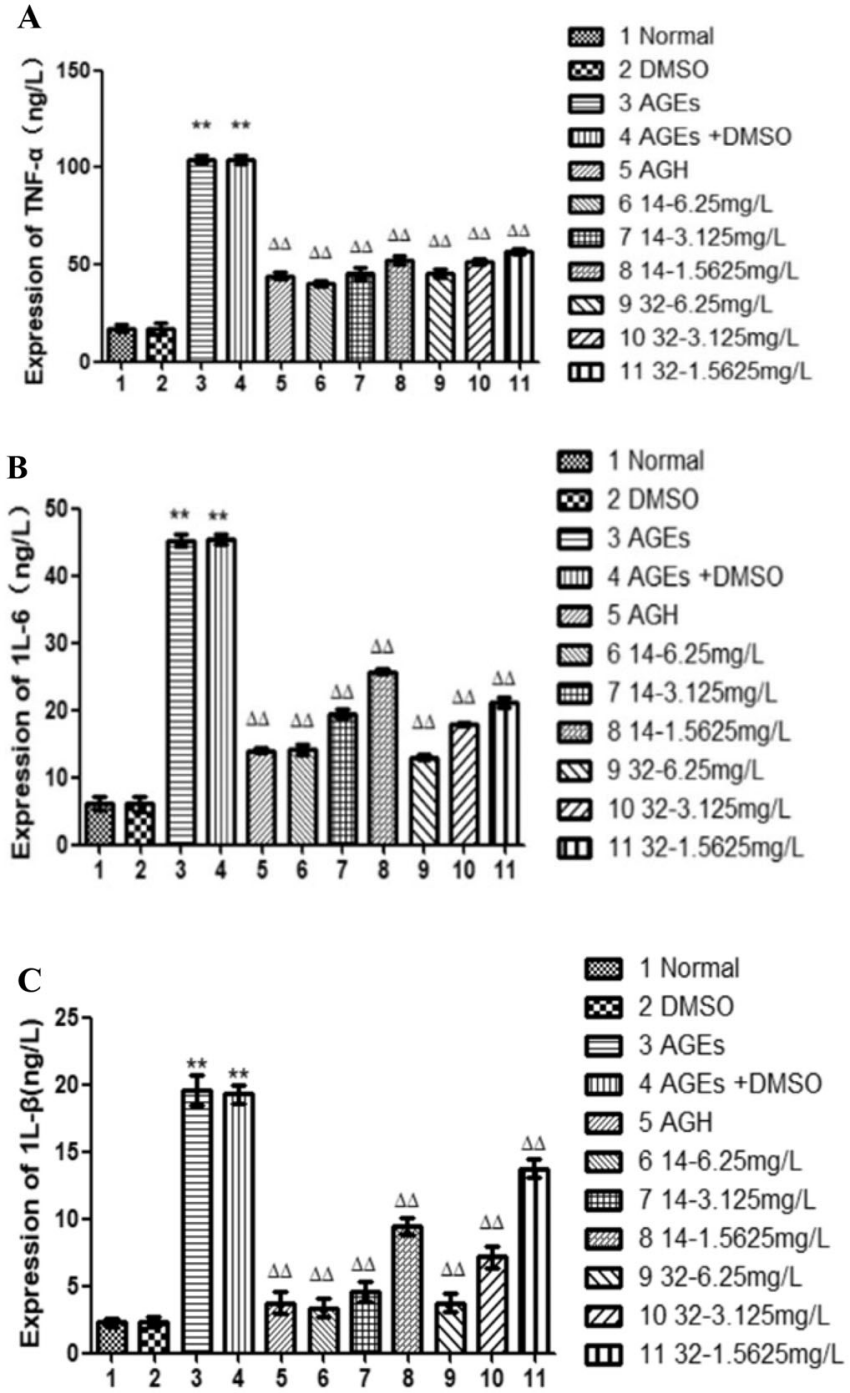
The influence of different concentrations of **14** and **32** on the expression of inflammatory factors TNF-α, 1 L-6, and 1 L-β.

Oxidative stress can cause inflammation^24^^,^^25^. Compared with that in the control group, the inflammatory cytokines TNF-α, 1 L-6, and 1 L-β were significantly increased in the cells of the AGEs group (*P* < 0.01), indicating that AGEs can induce inflammatory reactions in HASMCs. While, the expression of inflammatory cytokines TNF-α, 1 L-6, 1 L-β in the cells was progressively decreased by elevated concentrations of compounds **14** or **32** (*P* < 0.01), suggesting that compounds **14** and **32** can inhibit the inflammatory response of HASMCs in a concentration-dependent manner (Figure 8).

### 3.9. Effects of compound 14 and 32 on the expression of Runx2 mRNA

**Figure 9.**
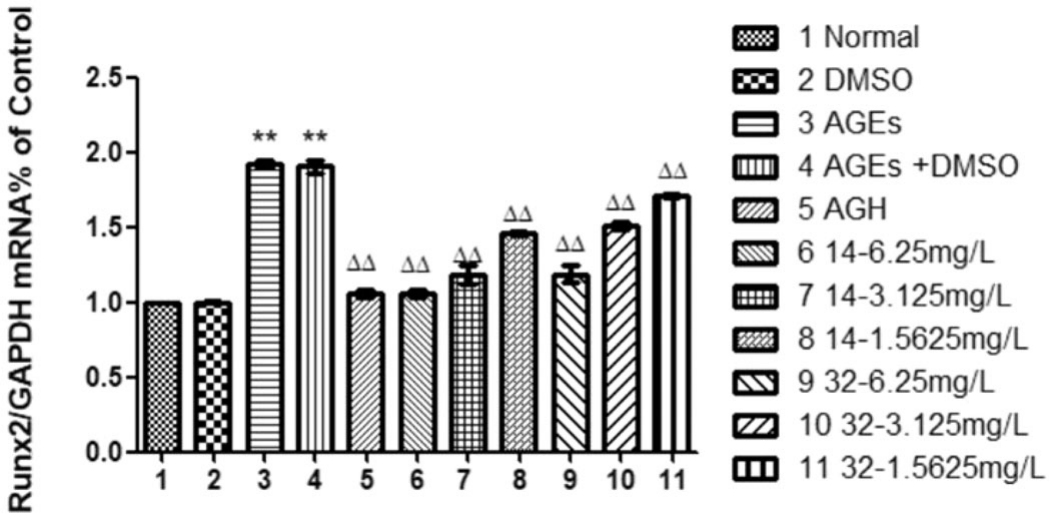
The effect of different concentrations of **14** and **32** on the expression of Runx2 mRNA.

Runt-related transcription factor 2 mRNA (Runx2 mRNA) is necessary for normal bone formation^26^, it was significantly increased by AGEs (*P* < 0.01), indicating that AGEs can induce osteogenic transformation of HASMCs. Compared with that in the AGEs group, the expression of Runx2 mRNA in the cells was progressively decreased by at elevated concentrations of compounds **14** or **32** (*P* < 0.01), suggesting that compounds **14** and **32** can inhibit the osteogenic transformation of HASMCs induced by AGEs in a concentration-dependent manner (Figure 9).

### 3.10. Effects of compound 14 on ERK1/2, PKC, NF-κB, RAGE, SM22-α, OPN, PPAR-γ protein expression induced by AGEs

**Figure 10.**
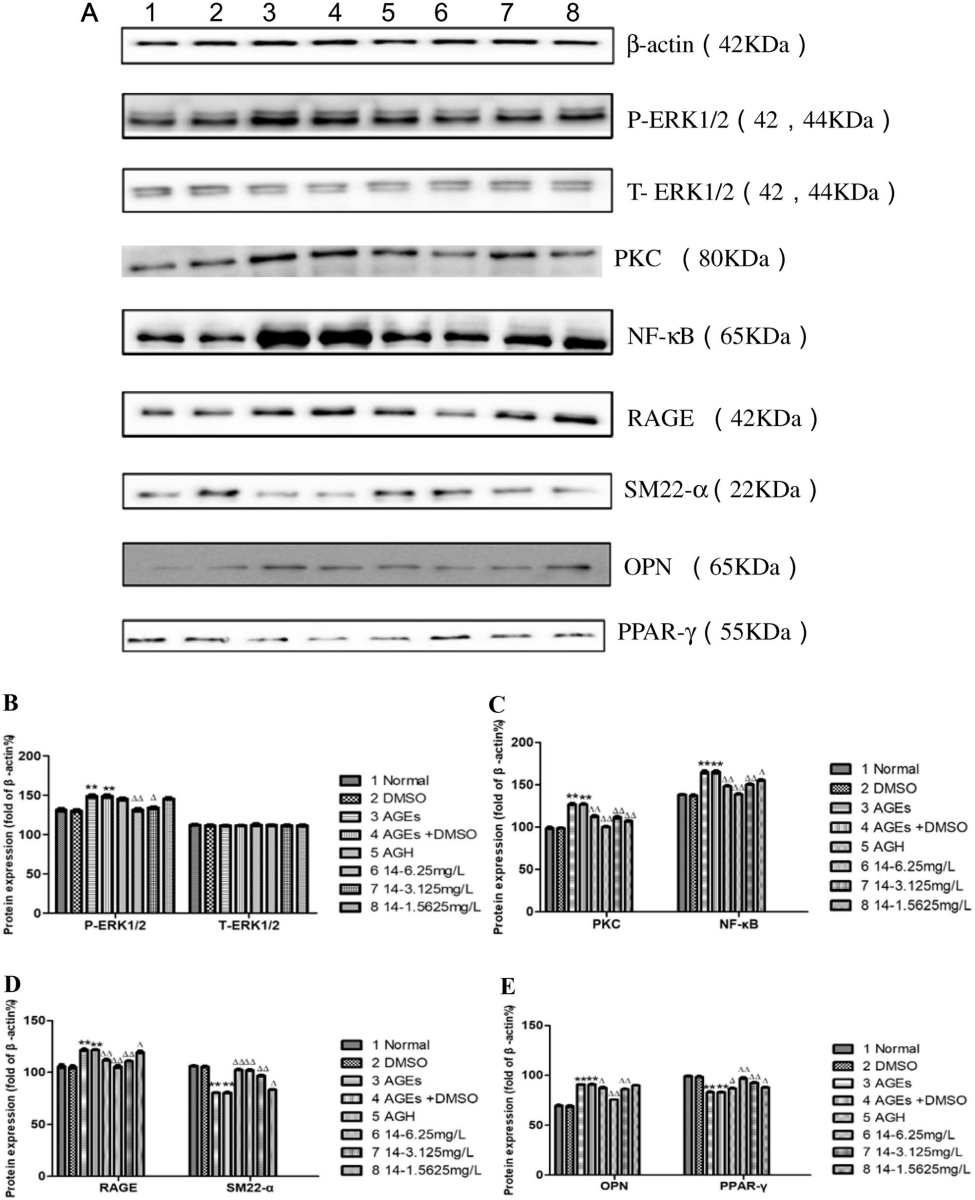
Effects of **14** at different concentrations on ERK1/2, PKC, NF-κB, RAGE, SM22-α, OPN, and PPAR-γ protein expression induced by AGEs.

Compared with that in the control group, the expression of P-ERK1/2, PKC, NF-κB, RAGE and OPN proteins in the AGEs group was significantly increased, the expression of at the same time SM22-α and PPAR-γ proteins were reduced (*P* < 0.01). As the concentration of compound **14** increases, the expression levels of P-ERK1/2, PKC, NF-κB, RAGE and OPN proteins gradually decreased, and the protein expression levels of SM22-α and PPAR-γ gradually increased, suggesting that compounds **14** can inhibit the related pathways of the differentiation of HASMCs into osteoblasts induced by AGEs in a concentration-dependent manner. We speculate that this type of compound can inhibit the oxidative stress process, thereby inhibiting NF-κB and PKC signalling pathways and reducing the inflammatory response of cells, ultimately reducing the calcification process (Figure 10).

The stimulation of AGEs increases the expression of RAGE^27^, and the AGEs/RAGE pathway is closely related to vascular calcification^28–30^. As the concentration of compound **14** increases, the expression of AGEs and RAGE is gradually inhibited. We inferred that it may promote the lysis of AGEs and down-regulate the expression of RAGE receptors and inhibit downstream signalling pathways and a variety of signal transduction proteins, including PKC, NF-κB, ERK1/2 to reduce AGEs-induced HASMCs osteoblast differentiation.

PPAR-γ is closely related to vascular calcification and has an important protective effect on the cardiovascular system. Studies have shown that PPAR-γ agonists can restore the expression of HASMCs surface markers^18^^,^^31^. Compound **14** obviously activated PPAR-γ in the calcification model in a concentration-dependent manner, which may be another way for compound **14** to protect HASMCs.

### 3.11. Molecular docking of compounds 14, 32 and BSA

**Figure 11.**
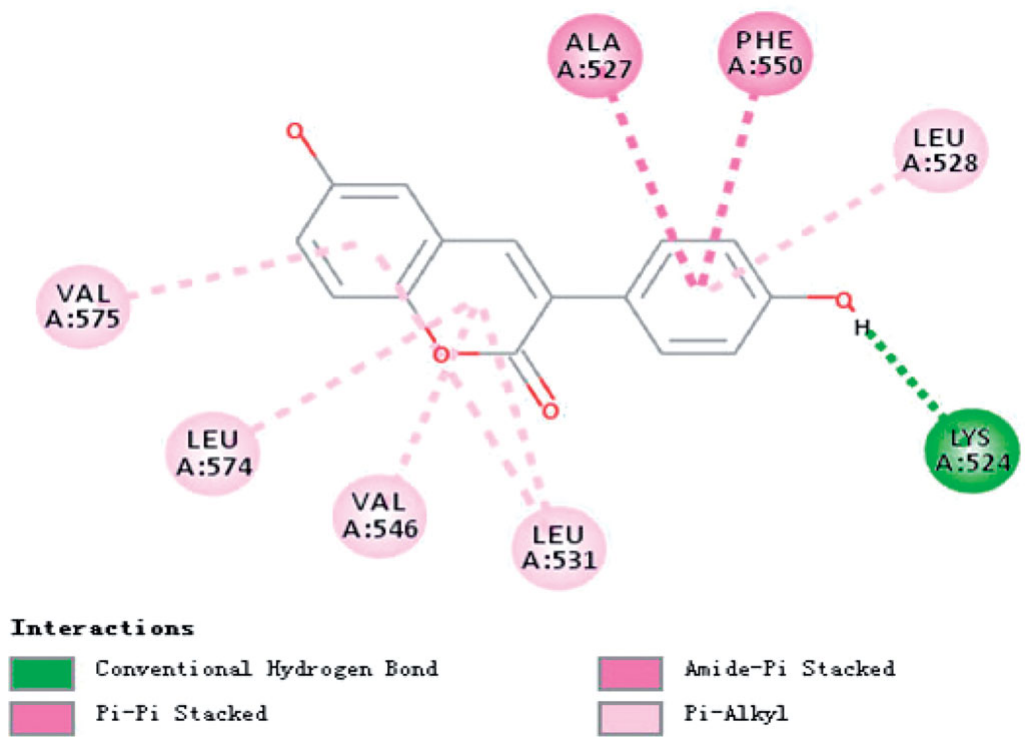
Research on molecular docking of compounds **14** and BSA.

Molecular docking can be used to reveal the interaction between compounds **14**, **32** and BSA. We, therefore, performed docking calculations for compound **14** (Figure 11) to LEU-574, VAL-546, LEU-531 in the binding pocket and the ligand A ring form Pi-Alkyl, where LEU-531 can also form Pi-Alkyl with the ligand B ring. VAL-575 can also interact with ligand B The ring forms Pi-Alkyl, while LEU-528 can form Pi-Alkyl with the ligand C ring, PHE-550, ALA-527 can form Amide-Pi Stacked and Pi-Pi Stacked with the ligand C ring, LYS-524 and ligand. The hydroxyl group on the C ring forms a conventional hydrogen bond.

**Figure 12.**
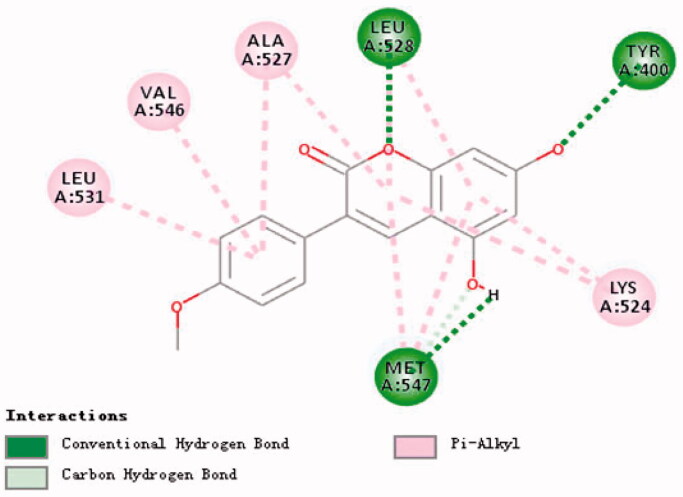
Research on molecular docking of compounds **32** and BSA.

The docking calculation result of compound **32** (Figure 12) shows that MET-547 in the binding pocket can form Pi-Alkyl with ligand A ring and ligand B ring, and form Conventional Hydrogen Bond, LEU with the hydroxyl group on ligand B ring −528 can form a Conventional Hydrogen Bond with the oxygen on the ligand A ring, and form Pi-Alkyl with the ligand B ring, TYR-400 can form a Conventional Hydrogen Bond with the hydroxyl group on the ligand B ring, LYS-524 can form a Conventional Hydrogen Bond with the ligand The A ring and the B ring form Pi-Alkyl. LEU-531, VAL-546, ALA-527 can form Pi-Alkyl with ligand C, and ALA-527 can also form Pi-Alkyl with ligand A.

## 4 Conclusion

In conclusion, the protective mechanism of compound **14** on calcification may be related to its antioxidant properties, inhibition of AGEs/RAGE, AKT/PKC, NF-κB, p38/ERK1/2 pathways and activation of PPAR-γ pathway, and also triggered the recovery of the expression of smooth muscle marker proteins SMA-α and SM22α and the down-regulation of OPN expression, a key osteogenic factor, which ultimately delayed the calcification transition process of HUVSMCs.

Compound **14** is validated at the molecular and cellular levels and is a potential drug for the treatment of vascular calcification, and in subsequent studies, we will focus on finding active derivatives for animal and human experiments.

**Table 1.** Main English abbreviations.

Abbreviations	Detailed explanation
ALP	Alkaline phosphatase
AGE	Advanced glycation end products
ROS	Reactive oxygen species
SOD	Superoxide dismutase
AGH	Aminoguanidine hydrochloride
RAGE	Recombinant Receptor For Advanced Glycation Endproducts
NF-κB	Nuclear factor kappa-B
OPN	Osteopontin
PPAR-γ	Peroxisome proliferator-activated receptor γ
SM22-α	Smooth muscle 22-α
HASMCs	Human aortic smooth muscle cells
DMEM	Dulbecco's modified eagle medium
BSA	Bovine serum albumin
ERK	Extracellular regulated protein kinases
TNF-α	Tumor necrosis factor-α

## References

[1] Wang C, Tang Y, Wang Y, et al. Label-free quantitative proteomics identifies Smarca4 is involved in vascular calcification. Renal Failure 2019;41:220–8. 30973285 10.1080/0886022X.2019.1591997 PMC6461080

[2] Abbasian N. Vascular calcification mechanisms: updates and renewed insight into signaling pathways involved in high phosphate-mediated vascular smooth muscle cell calcification. Biomedicines 2021;9:804. 34356868 10.3390/biomedicines9070804 PMC8301440

[3] Schalkwijk C, Stehouwer C. Methylglyoxal, a highly reactive dicarbonyl compound, in diabetes, its vascular complications, and other age-related diseases. Physiol Rev 2020;100:407–61. 31539311 10.1152/physrev.00001.2019

[4] Masbuchin AN, Rohman MS, Liu P-Y. Role of glycosylation in vascular calcification. Int J Mol Sci 2021;22:9829. 34575990 10.3390/ijms22189829 PMC8469761

[5] Zhang X, Chen J, Wang S. Serum amyloid A induces a vascular smooth muscle cell phenotype switch through the p38 MAPK signaling pathway. BioMed Res Int 2017;2017:4941379. 28642873 10.1155/2017/4941379 PMC5469989

[6] Lacolley P, Regnault V, Segers P, Laurent S. Vascular smooth muscle cells and arterial stiffening: relevance in development, aging, and disease. Physiol Rev 2017;97:1555–617. 28954852 10.1152/physrev.00003.2017

[7] Zazzeroni L, Faggioli G, Pasquinelli G. Mechanisms of arterial calcification: the role of matrix vesicles. Eur J Vasc Endovasc Surg 2018;55:425–32. 29371036 10.1016/j.ejvs.2017.12.009

[8] Lei Y, Sinha A, Nosoudi N, et al. Hydroxyapatite and calcified elastin induce osteoblast-like differentiation in rat aortic smooth muscle cells. Exp Cell Res 2014;323:198–208. 24447384 10.1016/j.yexcr.2014.01.011 PMC3969787

[9] Mazzone A, Clemente A, Sbrana S, et al. Statins association with calcification in coronary plaque and heart valves: a possible different clinical significance: Montignoso HEart and Lung Project (MHELP) study preliminary data in primary cardiovascular prevention. Eur J Prev Cardiol 2021;28:e15–e17. 32539450 10.1177/2047487320932330

[10] Al Rifai M, Blaha MJ, Patel J, et al. Coronary artery calcification, statin use and long-term risk of atherosclerotic cardiovascular disease events (from the multi-ethnic study of atherosclerosis). Am J Cardiol 2020;125:835–9. 31980142 10.1016/j.amjcard.2019.12.031

[11] Saisho Y. Metformin and inflammation: its potential beyond glucose-lowering effect. Endocr Metab Immune Disord Drug Targets. 2015;15:196–205. 25772174 10.2174/1871530315666150316124019

[12] Potu BK, Bhat KM, Rao MS, et al. Petroleum ether extract of Cissus quadrangularis (Linn.) enhances bone marrow mesenchymal stem cell proliferation and facilitates osteoblastogenesis. Clinics 2009;64:993–8. 19841707 10.1590/S1807-59322009001000010 PMC2763075

[13] Won KJ, Lee KP, Baek S, et al. Desalted Salicornia europaea extract attenuated vascular neointima formation by inhibiting the MAPK pathway-mediated migration and proliferation in vascular smooth muscle cells. Biomed Pharmacother 2017;94:430–8. 28778046 10.1016/j.biopha.2017.07.108

[14] Li W, Zhi W, Liu F, et al. nhibits VSMCs proliferation and migration by arresting cell cycle and activating HO-1 through MAPKs and NF-κB pathway. Int Immunopharmacol 2018;54:103–11. 29121532 10.1016/j.intimp.2017.10.017

[15] Lichota A, Gwozdzinski L, Gwozdzinski K. Therapeutic potential of natural compounds in inflammation and chronic venous insufficiency. Eur J Med Chem 2019;176:68–91. 31096120 10.1016/j.ejmech.2019.04.075

[16] Giordano E, Dávalos A, Crespo MC, et al. Soy isoflavones in nutritionally relevant amounts have varied nutrigenomic effects on adipose tissue. Molecules 2015;20:2310–22. 25647572 10.3390/molecules20022310 PMC6272387

[17] Hu Y, Wang B, Yang J, et al. Synthesis and biological evaluation of 3-arylcoumarin derivatives as potential anti-diabetic agents. J Enzyme Inhib Med Chem 2019;34:15–30. 30362362 10.1080/14756366.2018.1518958 PMC6211316

[18] Cartledge J, Eardley I, Morrison J. Nitric oxide‐mediated corpus cavernosal smooth muscle relaxation is impaired in ageing and diabetes. BJU Int 2001;87:402–7. 11251538 10.1046/j.1464-410x.2001.00065.x

[19] Hu C-T, Shao Y-D, Liu Y-Z, et al. Oxidative stress in vascular calcification. Clinica Chimica Acta 2021;519:101–10. 10.1016/j.cca.2021.04.012 33887264

[20] Zhu D, Mackenzie NCW, Shanahan CM, et al. BMP‐9 regulates the osteoblastic differentiation and calcification of vascular smooth muscle cells through an ALK 1 mediated pathway. J Cell Mol Med 2015;19:165–74. 25297851 10.1111/jcmm.12373 PMC4288360

[21] Karwowski W, Naumnik B, Szczepański M, Myśliwiec M. The mechanism of vascular calcification–a systematic review. Med Sci Monit 2012;18:RA1–R11. 22207127 10.12659/MSM.882181 PMC3560673

[22] Liu Y, Wang W-M, Zhang X-L, et al. AGE/RAGE promotes the calcification of human aortic smooth muscle cells via the Wnt/β-catenin axis. Am J Transl Res 2016;8:4644–56. 27904668 PMC5126310

[23] Selvaraju V, Joshi M, Suresh S, et al. Diabetes, oxidative stress, molecular mechanism, and cardiovascular disease–an overview. Toxicol Mech Methods 2012;22:330–5. 22394340 10.3109/15376516.2012.666648

[24] Tang L, Xu Y. e, Wei Y, He X. Uric acid induces the expression of TNF-α via the ROS-MAPK‑NF‑κB signaling pathway in rat vascular smooth muscle cells. Mol Med Rep 2017;16:6928–33. 28901421 10.3892/mmr.2017.7405

[25] Chen G, Zhu X, Zou X, et al. Retrospective analysis of thyroid nodules by clinical and pathological characteristics, and ultrasonographically detected calcification correlated to thyroid carcinoma in South China. Eur Surg Res 2009;42:137–42. 19174609 10.1159/000196506

[26] Jain A, Anand‐Srivastava MB. Natriuretic peptide receptor‐C‐mediated attenuation of vascular smooth muscle cell hypertrophy involves Gqα/PLCβ1 proteins and ROS‐associated signaling. Pharmacol Res Perspect 2018;6:e00375. 10.1002/prp2.375 PMC5817836 29417757

[27] Nakajima Y, Inagaki Y, Hiroshima Y, et al. Advanced glycation end-products enhance calcification in cultured rat dental pulp cells. J Endod 2013;39:873–8. 23791254 10.1016/j.joen.2012.11.027

[28] Kay AM, Simpson CL, Stewart JA. The role of AGE/RAGE signaling in diabetes-mediated vascular calcification. J Diabetes Res 2016;2016:6809703. 27547766 10.1155/2016/6809703 PMC4980539

[29] Tanikawa T, Okada Y, Tanikawa R, Tanaka Y. Advanced glycation end products induce calcification of vascular smooth muscle cells through RAGE/p38 MAPK. J Vasc Res 2009;46:572–80. 19571577 10.1159/000226225

[30] Liu L, Liu Y, Zhang Y, et al. High phosphate-induced downregulation of PPARγ contributes to CKD-associated vascular calcification. J Mol Cell Cardiol 2018;114:264–75. 29197521 10.1016/j.yjmcc.2017.11.021

[31] Panagiotopoulos S, O'Brien RC, Bucala R, et al. Aminoguanidine has an anti-atherogenic effect in the cholesterol-fed rabbit. Atherosclerosis 1998;136:125–31. 9544739 10.1016/s0021-9150(97)00192-5

